# Supplementary material for: A global-temporal analysis on Phytophthora sojae resistance-gene efficacy
Source: Nat Commun. 2023 Sep 27;14:6043. doi: 10.1038/s41467-023-41321-7 (PMC10533513; doi:10.1038/s41467-023-41321-7)
Supplement: Supplementary file 1 — Supplementary Information [file 41467_2023_41321_MOESM1_ESM.pdf]

A global-temporal analysis of *Phytophthora sojae* resistance-gene efficacy

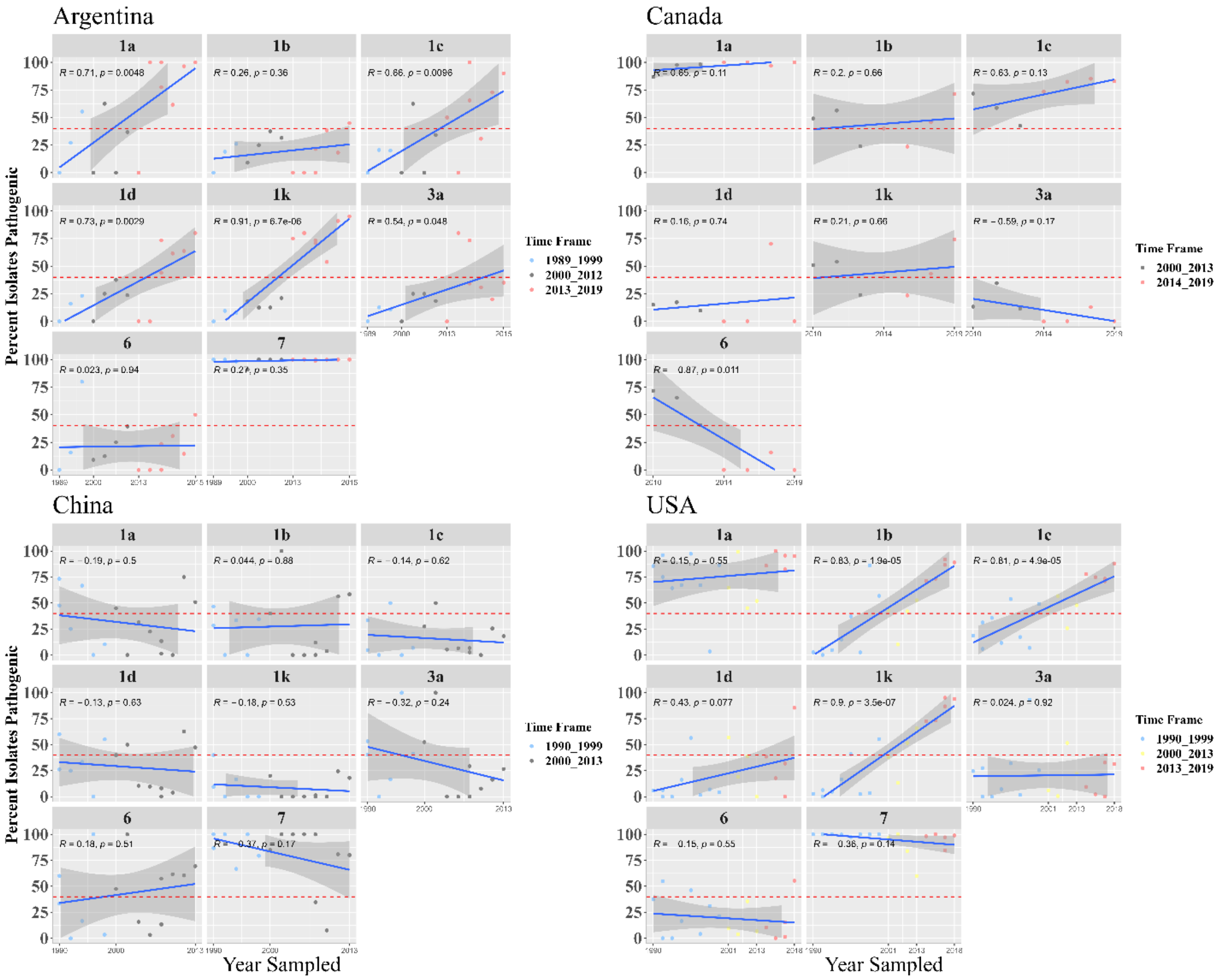


Supplementary Figure 1. Percent of isolates able to cause disease on each *Rps*-gene by country and year sampled. Red dotted line represents the 40% population management threshold. Dots represent percent of the samples from individual studies data, plotted by year sampled and colored by timeframes used in analysis. Pearson correlation coefficient and significance shown for each *Rps*-gene and nation. Linear regression and 95% confidence interval of the mean Rps gene efficacy over time represented as blue line and gray border, respectively. Source data are provided as a Source Data file.

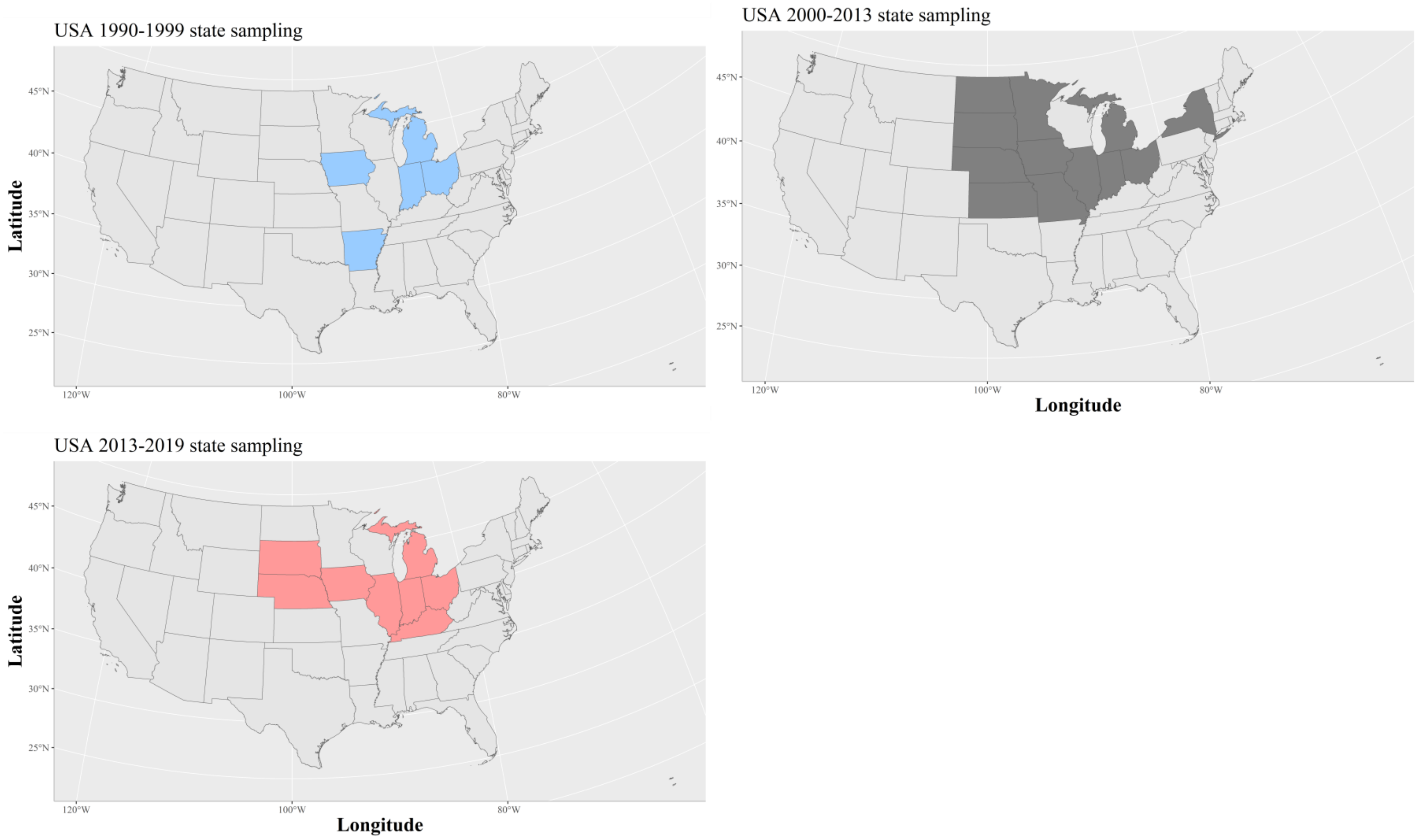


Supplementary Figure 2. Spatial-temporal sampling of the United States at each time frame studied. 1990-1999 state samplings are indicated in blue, 2000-2013 state samplings are indicated in black, 2013-2019 state samplings are indicated in red. Map shapefiles obtained from the Commission for Environmental Cooperation [1].

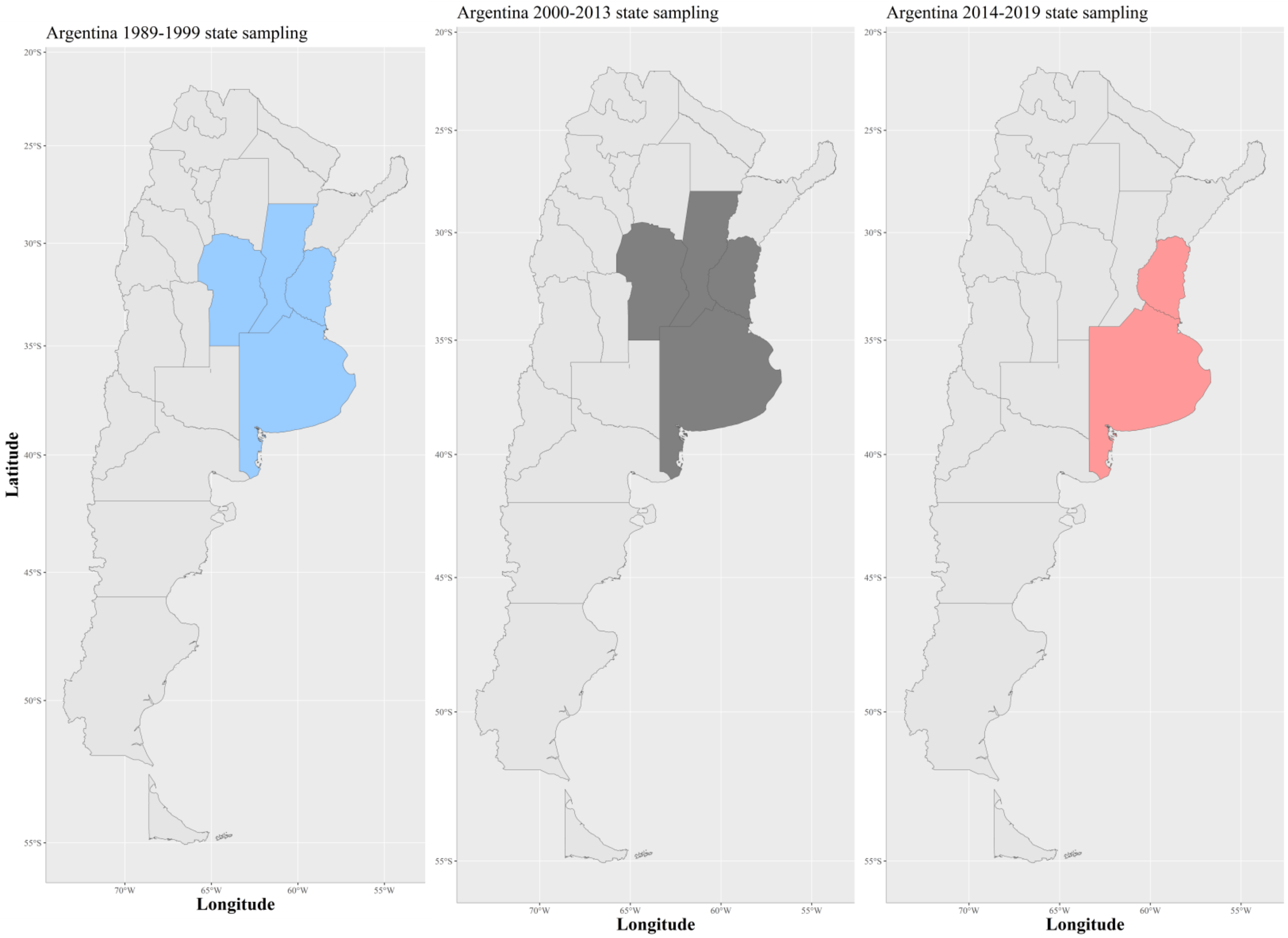

Argentina 1989-1999 state sampling
Argentina 2000-2013 state sampling
Argentina 2014-2019 state sampling
20°S
25°S
30°S
35°S
40°S
45°S
50°S
55°S
70°W
65°W
60°W
55°W
Latitude
Longitude

Supplementary Figure 3. Spatial-temporal sampling of Argentina at each time frame studied. 1989-1999 state samplings are indicated in blue, 2000-2013 state samplings are indicated in black, 2014-2019 state samplings are indicated in red. Map shapefile obtained from the Humanitarian Data Exchange [2].

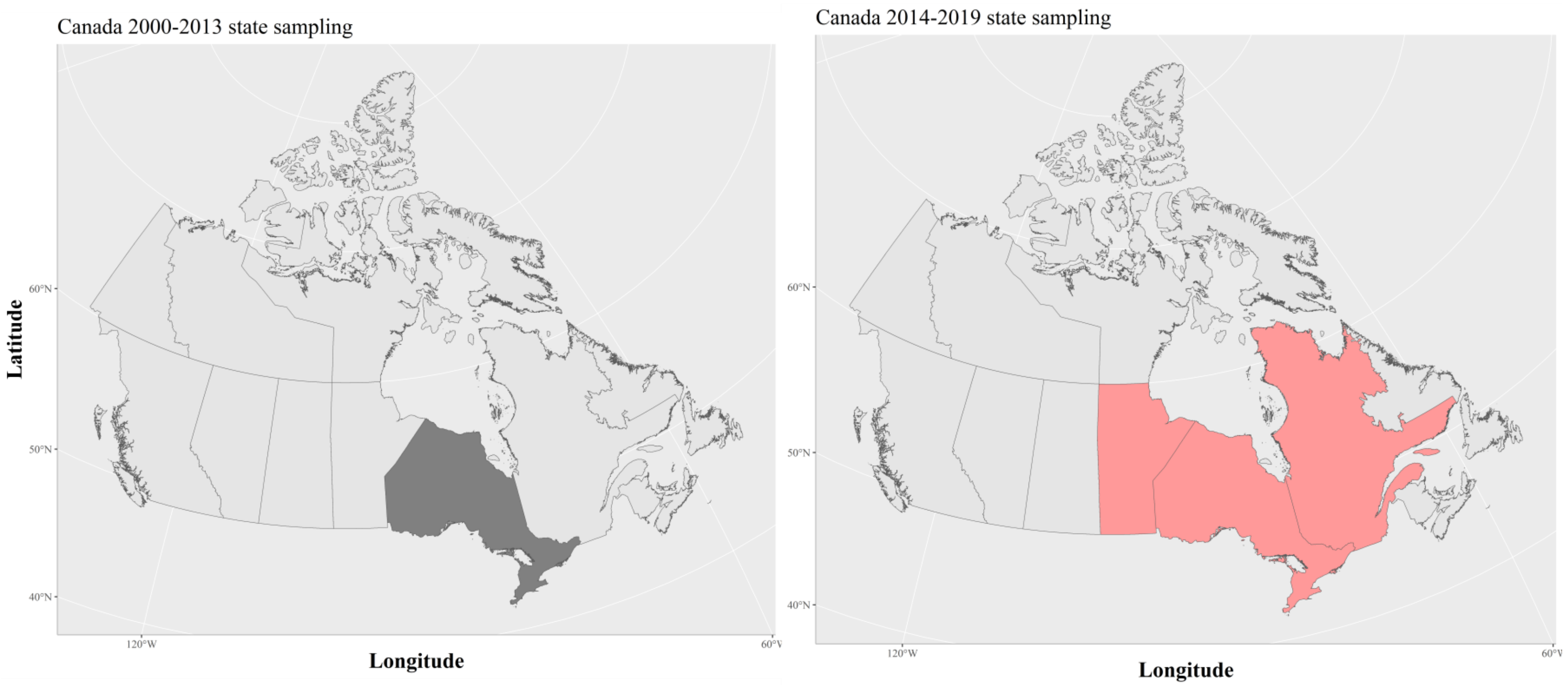


Supplementary Figure 4. Spatial-temporal sampling of Canada at each time frame studied. 2000-2013 state samplings are indicated in black and 2014-2019 state samplings are indicated in red. Map shapefiles obtained from the Commission for Environmental Cooperation [1].

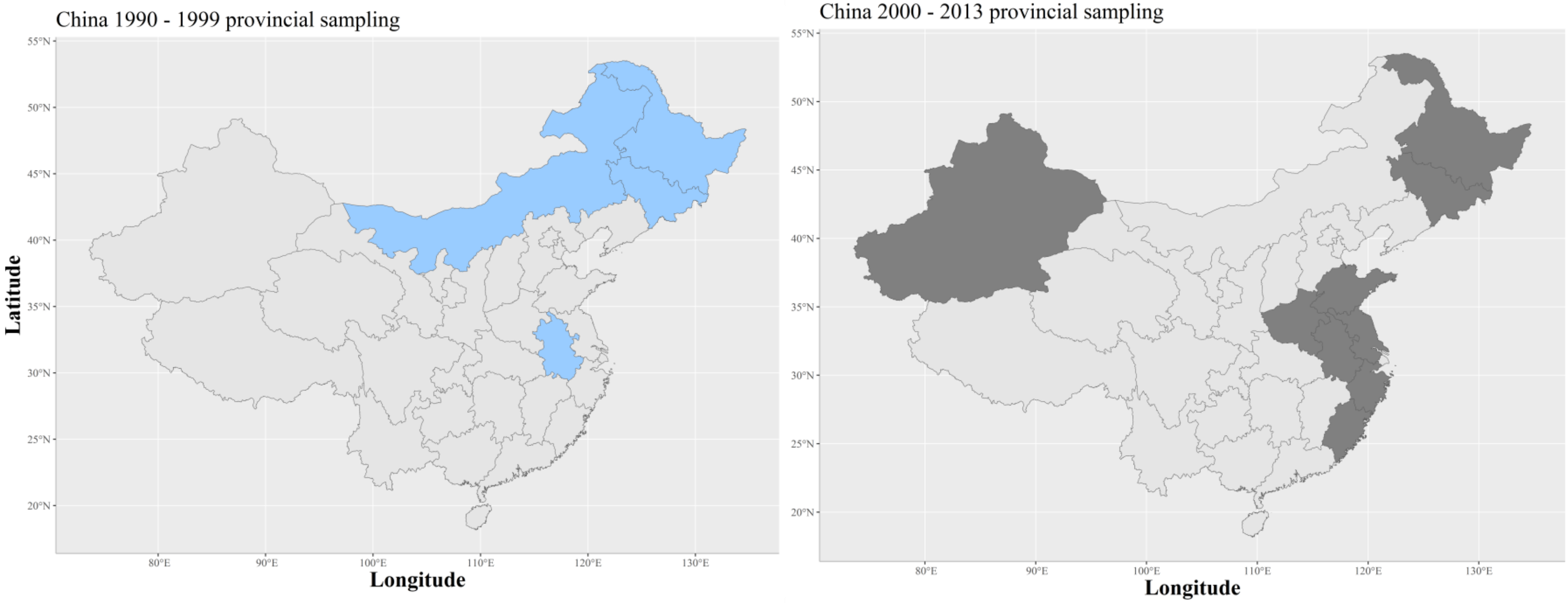


Supplementary Figure 5. Spatial-temporal sampling of China at each time frame studied. 1989-1999 provincial samplings are indicated in blue and 2000-2013 provincial samplings are indicated in black. Map shapefile obtained from the Humanitarian Data Exchange [3].

Supplementary Note 1 – Study Search Strategy

Google Scholar and Web of Science were used to identify *Phytophthora sojae* pathotype studies for integration into the Global *Phytophthora sojae* pathotype database and potential use within this systematic review. The authors used these search engines on September 21$^{st}$, 2021 to identify historic *P. sojae* pathotype surveys and again on June 6$^{th}$, 2022 for the most recent pathotype studies published. This supplement describes the search terms used within Google Scholar and Web of Science to identify studies published Prior to June 6$^{th}$, 2022.

The same search terms were used for Google Scholar and Web of Science on both September 21$^{st}$, 2021 and June 6$^{th}$, 2022. The search terms used were as follows:

Phytophthora sojae pathotype

Phytophthora sojae pathotype survey

Phytophthora sojae virulence survey

Phytophthora sojae Race

Supplementary Note 2. PRISMA 2020 Abstract checklist

| Section and Topic | Item # | Checklist item | Reported (Yes/No) |
|---|---|---|---|
| **TITLE** | | | |
| Title | 1 | Identify the report as a systematic review. | No |
| **BACKGROUND** | | | |
| Objectives | 2 | Provide an explicit statement of the main objective(s) or question(s) the review addresses. | Yes |
| **METHODS** | | | |
| Eligibility criteria | 3 | Specify the inclusion and exclusion criteria for the review. | Yes |
| Information sources | 4 | Specify the information sources (e.g. databases, registers) used to identify studies and the date when each was last searched. | Yes |
| Risk of bias | 5 | Specify the methods used to assess risk of bias in the included studies. | No |
| Synthesis of results | 6 | Specify the methods used to present and synthesise results. | No |
| **RESULTS** | | | |
| Included studies | 7 | Give the total number of included studies and participants and summarise relevant characteristics of studies. | Yes |
| Synthesis of results | 8 | Present results for main outcomes, preferably indicating the number of included studies and participants for each. If meta-analysis was done, report the summary estimate and confidence/credible interval. If comparing groups, indicate the direction of the effect (i.e. which group is favoured). | Yes |
| **DISCUSSION** | | | |
| Limitations of evidence | 9 | Provide a brief summary of the limitations of the evidence included in the review (e.g. study risk of bias, inconsistency and imprecision). | No |
| Interpretation | 10 | Provide a general interpretation of the results and important implications. | Yes |
| **OTHER** | | | |
| Funding | 11 | Specify the primary source of funding for the review. | No |
| Registration | 12 | Provide the register name and registration number. | No |

*From:* Page MJ, McKenzie JE, Bossuyt PM, Boutron I, Hoffmann TC, Mulrow CD, et al. The PRISMA 2020 statement: an updated guideline for reporting systematic reviews. BMJ 2021;372:n71. doi: 10.1136/bmj.n71

For more information, visit: http://www.prisma-statement.org/

Supplementary Note 3. PRISMA 2020 Manuscript checklist.

| Section and Topic | Item # | Checklist item | Reported on page # |
|---|---|---|---|
| **TITLE** | | | |
| Title | 1 | Identify the report as a systematic review. | **1** |
| **ABSTRACT** | | | |
| Abstract | 2 | See the PRISMA 2020 for Abstracts checklist. | **3** |
| **INTRODUCTION** | | | |
| Rationale | 3 | Describe the rationale for the review in the context of existing knowledge. | **6** |
| Objectives | 4 | Provide an explicit statement of the objective(s) or question(s) the review addresses. | **6** |
| **METHODS** | | | |
| Eligibility criteria | 5 | Specify the inclusion and exclusion criteria for the review and how studies were grouped for the syntheses. | **15-16** |
| Information sources | 6 | Specify all databases, registers, websites, organisations, reference lists and other sources searched or consulted to identify studies. Specify the date when each source was last searched or consulted. | **15** |
| Search strategy | 7 | Present the full search strategies for all databases, registers and websites, including any filters and limits used. | **Supplementary Note 1** |
| Selection process | 8 | Specify the methods used to decide whether a study met the inclusion criteria of the review, including how many reviewers screened each record and each report retrieved, whether they worked independently, and if applicable, details of automation tools used in the process. | **15** |
| Data collection process | 9 | Specify the methods used to collect data from reports, including how many reviewers collected data from each report, whether they worked independently, any processes for obtaining or confirming data from study investigators, and if applicable, details of automation tools used in the process. | **15** |
| Data items | 10a | List and define all outcomes for which data were sought. Specify whether all results that were compatible with each outcome domain in each study were sought (e.g. for all measures, time points, analyses), and if not, the methods used to decide which results to collect. | **15** |
| | 10b | List and define all other variables for which data were sought (e.g. participant and intervention characteristics, funding sources). Describe any assumptions made about any missing or unclear information. | **15** |
| Study risk of bias assessment | 11 | Specify the methods used to assess risk of bias in the included studies, including details of the tool(s) used, how many reviewers assessed each study and whether they worked independently, and if applicable, details of automation tools used in the process. | **15** |
| Effect measures | 12 | Specify for each outcome the effect measure(s) (e.g. risk ratio, mean difference) used in the synthesis or presentation of results. | **16-17** |
| Synthesis methods | 13a | Describe the processes used to decide which studies were eligible for each synthesis (e.g. tabulating the study intervention characteristics and comparing against the planned groups for each synthesis (item #5)). | **15-16** |
| | 13b | Describe any methods required to prepare the data for presentation or synthesis, such as handling of missing summary statistics, or data conversions. | **15-17** |
| | 13c | Describe any methods used to tabulate or visually display results of individual studies and syntheses. | **15-17** |
| | 13d | Describe any methods used to synthesize results and provide a rationale for the choice(s). If meta-analysis was performed, describe the model(s), method(s) to identify the presence and extent of statistical heterogeneity, and software package(s) used. | **15-17** |
| | 13e | Describe any methods used to explore possible causes of heterogeneity among study results (e.g. subgroup analysis, meta-regression). | **NA** |

| Section and Topic | Item # | Checklist item | Reported on page # |
|---|---|---|---|
| | 13f | Describe any sensitivity analyses conducted to assess robustness of the synthesized results. | **NA** |
| Reporting bias assessment | 14 | Describe any methods used to assess risk of bias due to missing results in a synthesis (arising from reporting biases). | **NA** |
| Certainty assessment | 15 | Describe any methods used to assess certainty (or confidence) in the body of evidence for an outcome. | **16-17** |
| **RESULTS** | | | |
| Study selection | 16a | Describe the results of the search and selection process, from the number of records identified in the search to the number of studies included in the review, ideally using a flow diagram. | **7** |
| | 16b | Cite studies that might appear to meet the inclusion criteria, but which were excluded, and explain why they were excluded. | **15** |
| Study characteristics | 17 | Cite each included study and present its characteristics. | **15** |
| Risk of bias in studies | 18 | Present assessments of risk of bias for each included study. | **Supplementary Table 1** |
| Results of individual studies | 19 | For all outcomes, present, for each study: (a) summary statistics for each group (where appropriate) and (b) an effect estimate and its precision (e.g. confidence/credible interval), ideally using structured tables or plots. | **8, 10, 11. Figures 1, 2, 3 and Tables 2 & 3** |
| Results of syntheses | 20a | For each synthesis, briefly summarise the characteristics and risk of bias among contributing studies. | **12** |
| | 20b | Present results of all statistical syntheses conducted. If meta-analysis was done, present for each the summary estimate and its precision (e.g. confidence/credible interval) and measures of statistical heterogeneity. If comparing groups, describe the direction of the effect. | **7-11, Tables 2 & 3** |
| | 20c | Present results of all investigations of possible causes of heterogeneity among study results. | **NA** |
| | 20d | Present results of all sensitivity analyses conducted to assess the robustness of the synthesized results. | **NA** |
| Reporting biases | 21 | Present assessments of risk of bias due to missing results (arising from reporting biases) for each synthesis assessed. | **12** |
| Certainty of evidence | 22 | Present assessments of certainty (or confidence) in the body of evidence for each outcome assessed. | **7-11** |
| **DISCUSSION** | | | |
| Discussion | 23a | Provide a general interpretation of the results in the context of other evidence. | **11-14** |
| | 23b | Discuss any limitations of the evidence included in the review. | **11-14** |
| | 23c | Discuss any limitations of the review processes used. | **12** |
| | 23d | Discuss implications of the results for practice, policy, and future research. | **14** |
| **OTHER INFORMATION** | | | |
| Registration and protocol | 24a | Provide registration information for the review, including register name and registration number, or state that the review was not registered. | **14-15** |
| | 24b | Indicate where the review protocol can be accessed, or state that a protocol was not prepared. | **14-15** |

| Section and Topic | Item # | Checklist item | Reported on page # |
|---|---|---|---|
| | 24c | Describe and explain any amendments to information provided at registration or in the protocol. | **NA** |
| Support | 25 | Describe sources of financial or non-financial support for the review, and the role of the funders or sponsors in the review. | **25** |
| Competing interests | 26 | Declare any competing interests of review authors. | **26** |
| Availability of data, code and other materials | 27 | Report which of the following are publicly available and where they can be found: template data collection forms; data extracted from included studies; data used for all analyses; analytic code; any other materials used in the review. | **17** |

*From:* Page MJ, McKenzie JE, Bossuyt PM, Boutron I, Hoffmann TC, Mulrow CD, et al. The PRISMA 2020 statement: an updated guideline for reporting systematic reviews. BMJ 2021;372:n71. doi: 10.1136/bmj.n71

For more information, visit: http://www.prisma-statement.org/

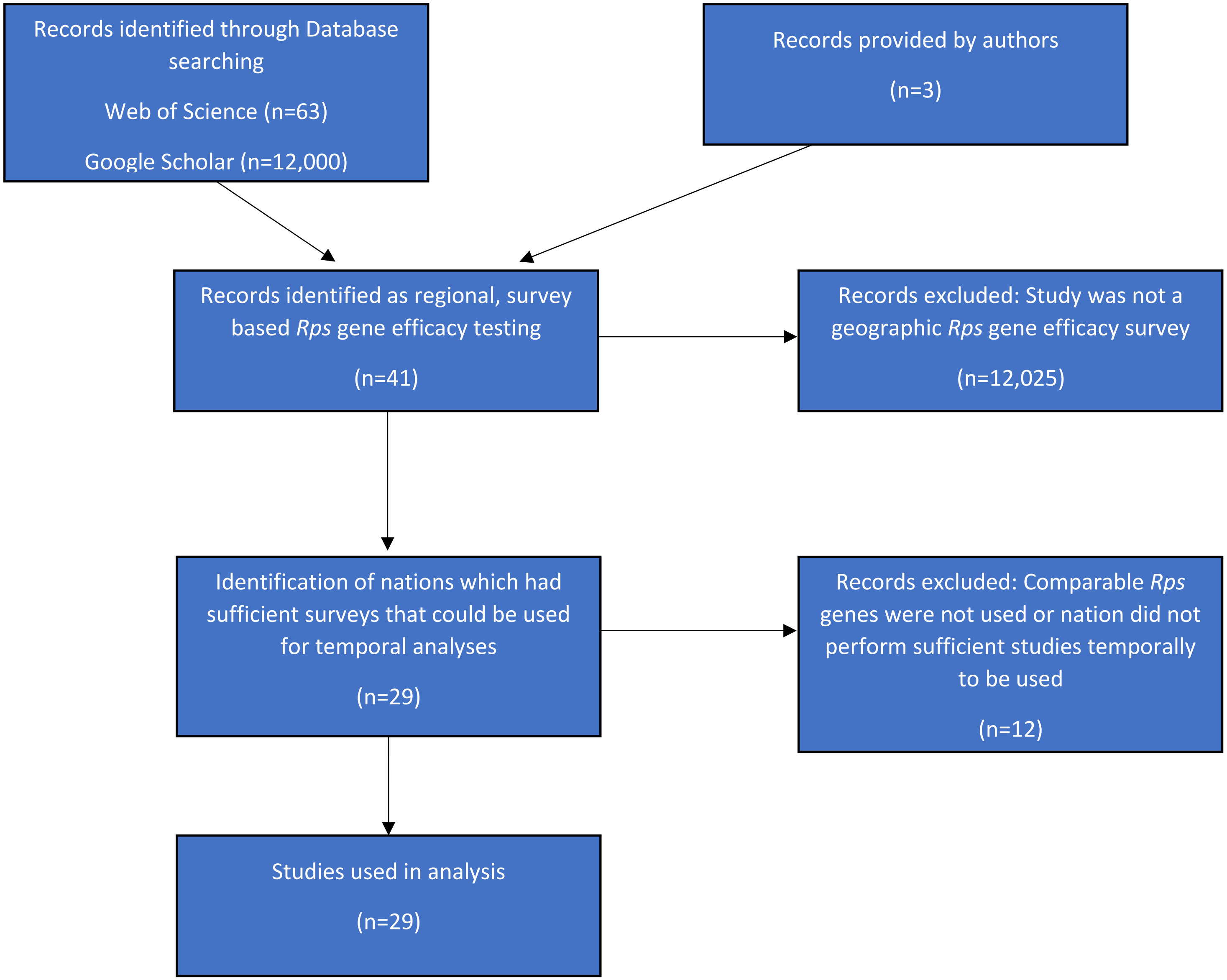


Supplementary Figure 6. PRISMA diagram. Flowchart depicts how studies were identified for use, or excluded, within this systematic review. Values within brackets are the number of identified studies which met the criteria for use in the manuscript at each step, or the number of studies which were excluded, respectively.

Supplementary Table 1. Risk of bias assessment among studies used in analysis.

| Study | Isolate recovery method | Pathoype evaluation method and environmental conditions | Overall Bias risk assessment |
|---|---|---|---|
| Schmitthenner et al 1994 | Medium risk* | low risk | Medium risk* |
| Yang et al 1996 | Medium risk* | low risk | Medium risk* |
| Abney et al 1997 | Medium risk* | low risk | Medium risk* |
| Kaitany et al 2001 | High risk** | low risk | High risk** |
| Dorrance et al 2003 | low risk | low risk | low risk |
| Jackson et al 2004 | low risk | low risk | low risk |
| Malvick and Grunden 2004 | low risk | low risk | low risk |
| Nelson et al 2008 | low risk | low risk | low risk |
| Robertson et al 2009 | low risk | low risk | low risk |
| Dorrance et al 2016 | Medium risk* | low risk | Medium risk* |
| Hebb et al 2022 | low risk | low risk | low risk |
| Chowdhury et al 2021 | low risk | low risk | low risk |
| McCoy et al 2022a | low risk | low risk | low risk |
| Matthiesen et al 2021 | Medium risk* | low risk | Medium risk* |
| Barreto et al 1995 | Medium risk* | low risk | Medium risk* |
| Grijalba et al 2014 | Medium risk* | low risk | Medium risk* |
| Grijalba et al 2020a | Medium risk* | low risk | Medium risk* |
| Grijalba et al 2020b | Medium risk* | low risk | Medium risk* |
| Xue et al 2015 | low risk | low risk | low risk |
| Henriquez et al 2020 | High risk** | low risk | High risk** |
| Tremblay et al 2021 | low risk | low risk*** | low risk |
| Jingzhi et al 2002 | High risk** | low risk | High risk** |
| Zhu et al 2003 | Medium risk* | low risk | Medium risk* |
| Xiuhong et al 2003 | Medium risk* | low risk | Medium risk* |
| Zhang et al 2010 | low risk | low risk | low risk |
| Cui et al 2010 | low risk | low risk | low risk |
| Linkai et al 2012 | low risk | low risk | low risk |

| | | | |
|---|---|---|---|
| Tian et al 2016 | low risk | low risk | low risk |
| Zhang et al 2021 | low risk | low risk | low risk |

Risk is defined here as potential to bias results either through artificial selection of virulent isolates (isolation from field plant which potentially contain *Rps* genes) or through pathotype evaluation methods and conditions which could skew results.
* some field plant samples were used for isolations, in addition to soil baiting, to recover isolates to testing
** only field plant isolations were used to recover isolates for testing
*** Tremblay et al used a qPCR assay for *P. sojae Avr* genes for pathotype testing. While the assay has been shown to be consistent with other pathotyping methods, it is the only current study which used these methods

Supplementary information references

1. Commission for Environmental Cooperation (CEC). "North American Atlas – Political Boundaries". Statistics Canada, United States Census Bureau, Instituto Nacional de Estadística y Geografía (INEGI). Ed. 3.0, Vector digital data [1:10,000,000]. Available at http://www.cec.org/north-american-environmental-atlas/political-boundaries-2021/ (2022)
2. Humanitarian Data Exchange. "Argentina – Subnational Administrative Boundaries". *Humanitarian Data Exchange*. Retrieved April 17th, 2023. Available at https://data.humdata.org/dataset/cod-ab-arg
3. Humanitarian Data Exchange. "China – Subnational Administrative Boundaries". *Humanitarian Data Exchange*. Retrieved April 17th, 2023. Available at https://data.humdata.org/dataset/cod-ab-chn
4. Schmitthenner, A.F., Hobe, M., Bhat, R.G.. *Phytophthora sojae* Races in Ohio over a 10-year interval. *Plant Dis.* 78, 269–276 (1994).
5. Yang, X.B., Ruff, R.L., Meng, X.Q., Workneh, F.. Races of *Phytophthora sojae* in Iowa Soybean Fields. *Plant Dis.* **80**, 1418–1420 (1996).

6. Abney, T.S., Melgar, J.C., Richards, T.L., Scott, D.H., Grogan, J., Young, J.. New races of *Phytophthora sojae* with *Rps*1-d virulence. *Plant Dis.* **81**, 653–655 (1997).
7. Kaitany, R.C., Hart, L.P., Safir, G.R.. Virulence Composition of *Phytophthora sojae* in Michigan. *Plant Dis.* **85**, 1103–1106 (2001).
8. Dorrance, A., McClure, S., DeSilva, A.. Pathogenic Diversity of *Phytophthora sojae* in Ohio Soybean Fields. *Plant Dis.* **87**, 139–146 (2003).
9. Jackson, T.A., Kirkpatrick, T.L., Rupe, J.C.. Races of *Phytophthora sojae* in Arkansas Soybean Fields and Their Effects on Commonly Grown Soybean Cultivars. *Plant Dis.* **88**, 345–351 (2004).
10. Malvick, D.K., Grunden, E.. Traits of Soybean-Infecting Phytophthora Populations from Illinois Agricultural Fields. *Plant Dis*. **88**, 1139-1145.
11. Nelson, B.D., Mallik, I., Mcewen, D., Christianson, T.. Pathotypes, distribution, and metalaxyl sensitivity of *Phytophthora sojae* from North Dakota. *Plant Dis.* **92**, 1062–1066 (2008).
12. Robertson, A.E., Cianzio, S.R., Cerra, S.M., Pope, R.O.. Within-field Pathogenic Diversity of *Phytophthora sojae* in Commercial Soybean Fields in Iowa. *Plant Heal. Prog.* **10**, 8 (2009).
13. Dorrance, A.E., Kurle, J., Robertson, A.E., Bradley, C.A., Giesler, L., Wise, K., Concibido, V.C.. Pathotype Diversity of *Phytophthora sojae* in Eleven States in the United States. *Plant Dis.* **100**, 1429–1437 (2016).
14. Hebb, L.M., Bradley, C.A., Mideros, S.X., Telenko, D.E.P., Wise, K., Dorrance, A.E.. Pathotype Complexity and Genetic Characterization of *Phytophthora sojae* Populations in Illinois, Indiana, Kentucky, and Ohio. *Phytopathology*. **112**, 663-681 (2022).
15. Chowdhury, R.N., Tande, C., Byamukama, E.. Common *Phytophthora sojae* Pathotypes Occurring In South Dakota. *Plant Heal. Prog.* **22**, 1–6 (2021).
16. McCoy, A.G., Noel, Z.A., Jacobs, J.L., Clouse, K.M., Chilvers, M.I.. *Phytophthora sojae* Pathotype Distribution and Fungicide Sensitivity in Michigan. *Plant Dis.* **106**, 425–431 (2022).

17. Matthiesen, R.L., Schmidt, C., Garnica, V.C., Giesler, L.J., Robertson, A.E.. Comparison of *Phytophthora sojae* Populations in Iowa and Nebraska to Identify Effective *Rps* Genes for Phytophthora Stem and Root Rot Management. *Plant Heal. Prog.* **22**, 1–9 (2021).
18. Barreto, D., Stegman de Gurfinkel, B., Fortugno, C.. Races of *Phytophthora sojae* in Argentina and Reaction of Soybean Cultivars. *Plant Dis.* **75**, 599–600 (1995).
19. Grijalba, P.E., Gally, M.E.. Virulence of *Phytophthora sojae* in the Pampeana Subregion of Argentina from 1998 to 2004. *J. Phytopathol.* **163**, 723–730 (2014).
20. Grijalba, P.E., del C. Ridao, A., Guillin, E., Steciow, M.. Pathogenic diversity of *Phytophthora sojae* in the southeast of the Province of Buenos Aires. *Trop. Plant Pathol.* **45**, 397–401 (2020a).
21. Grijalba, P.E., Martínez, M.C., Guillin, E.. Pathotype and SSR variation in *Phytophthora sojae* from the Argentinean Pampas. *J. Phytopathol.* **168**, 228–243 (2020b).
22. Xue, A.G., Marchand, G., Chen, Y., Zhang, S., Cober, E.R., Tenuta, A.. Races of *Phytophthora sojae* in Ontario, Canada, 2010-2012. *Can. J. Plant Pathol.* **37**, 376–383 (2015).
23. Henriquez, M.A., Kim, Y.M., McLaren, D.L., Conner, R.L., Xue, A., Marchand, G., Yu, K., Chang, K.F., Hwang, S.F., Strelkov, S.E., Gossen, B.D.. First report on the pathotype diversity of *Phytophthora sojae* in Manitoba, Canada. *Crop Prot.* **137**, 1–10 (2020).
24. Tremblay, V., McLaren, D.L., Kim, Y.M., Strelkov, S., Conner, R., Wally, O., Bélanger, R.R.. Molecular assessment of pathotype diversity of *Phytophthora sojae* in Canada highlights declining sources of resistance in soybean. *Plant Dis.* **105**, 4006-4013 (2021).
25. Jingzhi, W., Hongyu, C.. Study on Pathogenic Differentiation of *Phytophthora sojae*. *Chinese J. oil Crop Sci.* **24**, 63–66 (2002).
26. Zhu, Z., Wang, H., Wang, X., Chang, R., Wu, X.. Distribution and virulence diversity of *Phytophthora sojae* in China. *Sci. Agric. Sin.* **36**, 793–799 (2003).
27. Xiuhong, X., Yiying, L., Juanjuan, C., Qingkai, Y.. Identification and physiological races of *Phytophthora sojae* and toxicity analysis. *Acta Phytophylacica Sin.* **30**, 125–128 (2003).

28. Zhang, S., Xu, P., Wu, J., Xue, A.G., Zhang, J., Li, W., Chen, C., Weiyuan Chen, Lv, H.. Races of *Phytophthora sojae* and their virulences on soybean cultivars in Heilongjiang, China. *Plant Dis.* **94**, 87–91 (2010).
29. Cui, L., Yin, W., Tang, Q., Dong, S., Zheng, X., Zhang, Z., Wang, Y.. Distribution, Pathotypes, and Metalaxyl Sensitivity of *Phytophthora sojae* from Heilongjiang and Fujian Provinces in China. *Plant Dis.* **95**, 881-884 (2010).
30. Linkai, C., Ynahong, H.. Study on the virulence composition of *Phytophthora sojae* in Xinjiang. *J. Henan Agric. Sci.* **41**, 88–90 (2012).
31. Tian, M., Zhao, L., Li, S., Huang, J., Sui, Z.. Pathotypes and metalaxyl sensitivity of *Phytophthora sojae* and their distribution in Heilongjiang, China 2011 – 2015. *J. Gen. Plant Pathol.* **82**, 132–141 (2016).
32. Zhang, Z., Zhao, Y., Yu, H., Chen, Y., Gu, X., Wen, J.. Pathotypes of *Phytophthora sojae* and their distribution in Jilin, China. *J. Plant Pathol.* **103**, 241–248 (2021).
