# Supplementary material for: A global-temporal analysis on Phytophthora sojae resistance-gene efficacy
Source: Nat Commun. 2023 Sep 27;14:6043. doi: 10.1038/s41467-023-41321-7 (PMC10533513; doi:10.1038/s41467-023-41321-7)
Supplement: Supplementary file 2 — Reporting Summary [file 41467_2023_41321_MOESM2_ESM.pdf]

nature portfolio

Corresponding author(s): Austin McCoy, Martin Chilvers

Last updated by author(s): 7/19/2023

Reporting Summary

Nature Portfolio wishes to improve the reproducibility of the work that we publish. This form provides structure for consistency and transparency in reporting. For further information on Nature Portfolio policies, see our Editorial Policies and the Editorial Policy Checklist.

Statistics

For all statistical analyses, confirm that the following items are present in the figure legend, table legend, main text, or Methods section.

n/a	Confirmed	
☐	☒	The exact sample size (*n*) for each experimental group/condition, given as a discrete number and unit of measurement
☐	☒	A statement on whether measurements were taken from distinct samples or whether the same sample was measured repeatedly
☐	☒	The statistical test(s) used AND whether they are one- or two-sided<br>*Only common tests should be described solely by name; describe more complex techniques in the Methods section.*
☒	☐	A description of all covariates tested
☒	☐	A description of any assumptions or corrections, such as tests of normality and adjustment for multiple comparisons
☐	☒	A full description of the statistical parameters including central tendency (e.g. means) or other basic estimates (e.g. regression coefficient) AND variation (e.g. standard deviation) or associated estimates of uncertainty (e.g. confidence intervals)
☐	☒	For null hypothesis testing, the test statistic (e.g. *F*, *t*, *r*) with confidence intervals, effect sizes, degrees of freedom and *P* value noted<br>*Give P values as exact values whenever suitable.*
☒	☐	For Bayesian analysis, information on the choice of priors and Markov chain Monte Carlo settings
☐	☒	For hierarchical and complex designs, identification of the appropriate level for tests and full reporting of outcomes
☐	☒	Estimates of effect sizes (e.g. Cohen's *d*, Pearson's *r*), indicating how they were calculated

Our web collection on statistics for biologists contains articles on many of the points above.

Software and code

Policy information about availability of computer code

Data collection	Phytophthora sojae pathotype studies were identified using Google Scholar and Web of Science on the 21st of September 2021 and searched again for studies published after the original search date on the 6th of June 2022. . Data from all identified P. sojae pathotype surveys were transcribed manually into Microsoft Excel® from published manuscripts or supplied by authors. Each survey data set was then validated using the 'hagis' R package (v3.1.4) for microbial phenotypic pathogenicity data to ensure the data collected accurately reflected what was reported in the published manuscripts. Studies which were not regional survey-based P. sojae pathotype studies on Rps gene efficacy were not used within this temporal analysis. A curated global P. sojae virulence phenotype database was established from all identified studies as of June 6th 2022 and published online (Zenodo accession #7850345).
Data analysis	R package software used in analysis, as well as versions used, are as follows: R version 4.1.1, 'hagis'(v3.1.4), 'ggplot2'(v3.3.5), 'vegan'(v2.5-7), 'ellipses'(v0.4.2), 'RVAideMemoire'(v0.9-81-2)

For manuscripts utilizing custom algorithms or software that are central to the research but not yet described in published literature, software must be made available to editors and reviewers. We strongly encourage code deposition in a community repository (e.g. GitHub). See the Nature Portfolio guidelines for submitting code & software for further information.

Data

Policy information about availability of data

All manuscripts must include a data availability statement. This statement should provide the following information, where applicable:

- Accession codes, unique identifiers, or web links for publicly available datasets
- A description of any restrictions on data availability
- For clinical datasets or third party data, please ensure that the statement adheres to our policy

The pathotype data used in this study, and all associate R code, has been deposited into the Zenodo database under accession number 7850345 (https://doi.org/10.5281/zenodo.7850345) and can be found on GitHub (https://github.com/AGmccoy/Phytophthora-sojae-global-pathotype-meta-analysis). The data are available under the Creative Commons Zero v1.0 Universal License. Source data are provided with this paper.

Human research participants

Policy information about studies involving human research participants and Sex and Gender in Research.

Reporting on sex and gender	NA
Population characteristics	NA
Recruitment	NA
Ethics oversight	NA

Note that full information on the approval of the study protocol must also be provided in the manuscript.

Field-specific reporting

Please select the one below that is the best fit for your research. If you are not sure, read the appropriate sections before making your selection.

☐ Life sciences ☐ Behavioural & social sciences ☒ Ecological, evolutionary & environmental sciences

For a reference copy of the document with all sections, see nature.com/documents/nr-reporting-summary-flat.pdf

Ecological, evolutionary & environmental sciences study design

All studies must disclose on these points even when the disclosure is negative.

Study description	In this 'first of its kind' study we conducted a global-temporal meta-analysis of Resistance genes (Rps genes) for management of Phytophthora sojae. We used phenotypic data from 29 studies, encompassing 5,121 Phytophthora sojae isolates, across three decades from four of the top ten soybean producing countries globally (Argentina, Canada, China, and the United States) to identify temporal changes in the efficacy of 8 soybean P. sojae resistance genes (Rps genes).
Research sample	time frames (i.e. 1990s, 200-2012, 2013-2019) were used to determine Rps gene efficacy across four contries (USA, Canada, Argentina, China). Previously conducted pathotype study data which was available was used in this meta-analysis. individual isolates of Phytophthora sojae were tested against soybean lines containing different single Rps genes in prior studies. Resistant or susceptible interactions were recorded and reported to identify effective Rps genes within each study and used for analysis in this study.
Sampling strategy	All identified (41) pathotype studies data were recorded into a public database. time frames were identified which could be used for a temporal meta-analysis in the USA, Argentina, Canada, and China. Other countries did not have sufficient temporal samplings, or used different resistance genes for testing.
Data collection	Phytophthora sojae pathotype studies were identified using Google Scholar and Web of Science on the 21st of September 2021 and searched again for studies published after the original search date on the 6th of June 2022. . Data from all identified P. sojae pathotype surveys were transcribed manually into Microsoft Excel® from published manuscripts or supplied by authors. Each survey data set was then validated using the 'hagis' R package for microbial phenotypic pathogenicity data to ensure the data collected accurately reflected what was reported in the published manuscripts. Studies which were not regional survey-based P. sojae pathotype studies on Rps gene efficacy were not used within this temporal analysis. A curated global P. sojae virulence phenotype database was established from all identified studies as of June 6th 2022 and published online (Zenodo accession #7850345).
Timing and spatial scale	Studies used in this systematic review collected data from 1989-2019 across four different countries soybean growing regions.
Data exclusions	Studies from countries which performed minimal pathotype surveys over time, or were outside of the time frames studied, were not used in analysis

Reproducibility	As this systematic review uses previously reported data, we used studies which reported adequate pathotype environmental conditions (methods). All code and data used in this meta-analysis has been made publicly available before publication.
Randomization	Isolates data was aggregated into 2-3 distinct time points based on the availability of data, for each country.
Blinding	NA

Did the study involve field work? ☐ Yes ☒ No

Reporting for specific materials, systems and methods

We require information from authors about some types of materials, experimental systems and methods used in many studies. Here, indicate whether each material, system or method listed is relevant to your study. If you are not sure if a list item applies to your research, read the appropriate section before selecting a response.

Materials & experimental systems

n/a	Involved in the study
☒	☐ Antibodies
☒	☐ Eukaryotic cell lines
☒	☐ Palaeontology and archaeology
☒	☐ Animals and other organisms
☒	☐ Clinical data
☒	☐ Dual use research of concern

Methods

n/a	Involved in the study
☒	☐ ChIP-seq
☒	☐ Flow cytometry
☒	☐ MRI-based neuroimaging
